# Short-term structured dietary and exercise interventions delay diabetes onset in prediabetic patients: a prospective quasi-experimental study

**DOI:** 10.3389/fendo.2025.1413206

**Published:** 2025-03-28

**Authors:** Zhen Wang, Li Qian, Jian-Tong Shen, Bing Wang, Xu-Hui Shen, Guo-Ping Shi

**Affiliations:** ^1^ School of Medicine and School of Nursing, Huzhou University, Huzhou, Zhejiang, China; ^2^ Huzhou Key Laboratory of Precise Prevention and Control of Major Chronic Diseases, Huzhou University, Huzhou, Zhejiang, China; ^3^ Department of General Practice, Huzhou City Longquan Street Huanzhu Community Health Service Center, Huzhou, Zhejiang, China; ^4^ School of Medicine, Southeast University, Nanjing, Jiangsu, China; ^5^ Department of Medicine, Brigham and Women's Hospital, Harvard Medical School, Boston, MA, United States

**Keywords:** prediabetes, diabetes, dietary intervention, exercise intervention, SAIDEs

## Abstract

**Hypothesis:**

Prediabetes indicates an increased risk of developing diabetes mellitus. We hypothesized that structured anti-inflammatory and antioxidant dietary and exercise interventions (SAIDEs) can reduce the onset of diabetes in prediabetic patients.

**Methods:**

This study included 542 prediabetic patients who met at least one of the three common criteria for prediabetes: fasting blood glucose (FBG), 2-h oral glucose tolerance (2h OGTT), or hemoglobin A1c (HbA1C). Patients were randomly assigned to one of four groups using the block randomization method: routine community intervention, dietary intervention, exercise intervention, or SAIDEs for 6 months. Follow-up assessments were conducted at 6 months and 7.5 years, monitoring diabetes-related outcomes, inflammatory markers, and diabetes progression.

**Results:**

At baseline, most tested variables, including age, gender, body weight, blood lipids, blood sugar, β-cell function, blood inflammatory and immunological markers, and energy intake, did not differ among the groups. After 6 months of short-term interventions (diet, exercise, and SAIDEs) and 6 months of follow-up, all intervention groups exhibited reduced total energy intake, body weight, blood pressure, blood cholesterol, and glucose levels, along with improved β-cell functions (all *p* < 0.001). Regardless of time considerations, intervention consistently increased total physical activity (*p* < 0.001). Short-term interventions also reduced blood IgE, high-sensitivity C-reactive protein, IL-6, and TNF-α, while increasing blood IL-4 and IL-10 (all *p* < 0.001). The prevalence of abnormal blood glucose markers—FBG, 2h OGTT, and HbA1C—significantly decreased within each intervention group after short-term intervention and 6 months of follow-up. The time-dependent Cox regression test did not indicate a significant effect of dietary or exercise intervention on diabetes incidence over the 8-year follow-up period. However, the log-rank test revealed significant differences in “survival” distribution among the four intervention groups (*χ* ^2^ = 15.63, *p* = 0.001). The mean survival time before diabetes onset was significantly longer in prediabetic patients who received SAIDEs than in those in other groups.

**Conclusions:**

Short-term intervention with SAIDEs exhibited significant anti-inflammatory activity and reduced the prevalence of abnormal blood glucose markers. These benefits persisted even after 6 months of follow-up. However, over the 8-year follow-up period, intensive SAIDEs did not reduce diabetes incidence among prediabetic patients but did delay its onset.

**Clinical trial registration:**

https://www.chictr.org.cn/searchproj.html, identifier ChiCTR-IOR-16008445.

## 1 Introduction

Prediabetes is an intermediate hyperglycemic state between normoglycemia and diabetes, classified as impaired fasting glucose (IFG), impaired glucose tolerance (IGT), or a combination of both (IFG and IGT) (1). The occurrence of prediabetes signifies an increased risk of developing diabetes mellitus, along with various macrovascular and microvascular complications, degenerative diseases, and tumors (1). Numerous studies have reported the presence of cardiovascular risk factors in patients with prediabetes or even at earlier stages (2–4). According to the natural history of diabetes, approximately 25% of individuals with prediabetes may develop type 2 diabetes (T2DM), around 25% may return to normal glucose levels, and nearly 50% may remain in the prediabetic state over the next 2 to 5 years (2–4). However, if lifestyle or drug intervention target major risk factors during the prediabetic stage, up to 50% of individuals with prediabetes may revert to normoglycemia (2–4).

The prevention of T2DM relies primarily on lifestyle modifications, including physical activity and dietary adjustments. The timing of intervention measures significantly influences their effectiveness (3). Preventive interventions for T2DM occur in two stages: an early stage targeting the general population, including healthy individuals, and a late stage focusing on high-risk groups, such as those with prediabetes, obesity, hypertension, and hyperlipidemia (3). Studies have shown that individuals’ responses to lifestyle interventions vary. Interventions targeting high-risk groups for T2DM were more effective than interventions targeting the entire population because of their awareness of health risks, beliefs in the benefits of interventions, and their compliance (3). For prediabetic patients, dietary choices, exercise, and other lifestyle modifications are generally recommended. Several representative lifestyle intervention studies have demonstrated the effectiveness of lifestyle interventions in reducing the risk of diabetes development from prediabetes, including the China Daqing Diabetes Prevention Study (CDQDPS) (5), the Finnish Diabetes Prevention Study (FIN-D2D) (6), and the American Diabetes Prevention Program (ADPP) (7). These interventional studies consistently confirmed the effects of short- and long-term lifestyle interventions in reducing the incidence of diabetes and cardiovascular and cerebrovascular diseases.

The role of inflammation, especially chronic low-grade inflammation in metabolic diseases, received increased attention in the past 20 years. Inflammation-targeted intervention or treatment in metabolic diseases remains the focus of the field (8–10). Functional foods contain bioactive components and are associated with physiological health benefits in the prevention and management of chronic diseases such as T2DM, including enhanced antioxidant and anti-inflammatory activities, insulin sensitivity, and anticholesterol functions. For instance, Mediterranean diets are naturally rich in polyphenols, terpenoids, flavonoids, alkaloids, sterols, pigments, and unsaturated fatty acids and serve as a model of functional foods. Some polyphenol-rich herbs, such as coffee, green tea, and black tea, also exert significant benefits similar to those of functional foods. Therefore, combining exercise with the intake of functional foods can stimulate and enhance multiple metabolic and cardiovascular protective effects (10).

Inflammatory molecules and immune factors are expressed at different stages of T2DM development, including high-sensitivity C-reactive protein (hs-CRP), immunoglobulin (Ig) E, tumor necrosis factor-α (TNF-α), interleukins (ILs), leptin, adiponectin, monocyte chemoattractant-1 (MCP-1), and plasminogen activator inhibitor-1 (PAI-1) (11–15). These proinflammatory biomarkers hold significant potential for predicting the occurrence and development of diabetes, as well as evaluating prevention strategies (11–15). In recent years, nutritionists have introduced the concept of an “anti-inflammatory diet” (10, 16, 17), a dietary approach designed to support optimal health. “Anti-inflammatory diet programs” provide a controlled energy structure with adequate vitamins, minerals, essential fatty acids, dietary fiber, and phytochemicals necessary for daily nutritional needs (10, 18, 19). However, most current diabetes diet intervention programs focus on single anti-inflammatory nutrients for managing prediabetes and T2DM. Few studies have integrated a structured anti-inflammatory and immunomodulatory diet combined with exercise interventions (SAIDEs) into daily dietary management. Additionally, intervention studies have often assessed efficacy using only a limited number of inflammatory markers, sometimes just a single indicator (10, 18, 19). Furthermore, growing evidence highlights a significant correlation between physical inactivity and chronic low-grade inflammation. Generally, moderate-intensity aerobic exercise plays a crucial role in preventing and managing T2DM and its complications (20–27). These benefits are primarily associated with improvements in anti-inflammatory conditions, oxidative stress reduction, and immune regulation (20–27). Based on the current knowledge from the dietary or exercise intervention programs, most of them evaluated the effects of intervention from the perspective of controlling blood sugar levels and traditional cardiovascular risk factors. Very few focused on the perspective of anti-inflammation and immunoregulation. Those anti-inflammation- and immunoregulation-oriented studies were cross-sectional studies on the relationship between self-reported physical activity and inflammation and immunity. There are currently no prospective studies evaluating the effects of exercise intervention using SAIDEs.

T2DM has become a major global public health issue. However, awareness of prediabetic status remains low among patients. It is essential for health service providers to strengthen disease awareness in individuals with prediabetes, equip them with diabetes management knowledge and skills, and promote compliance. Implementing SAIDEs to address key risk factors, such as an irregular diet and lack of exercise, is expected to improve chronic inflammation and delay—or even prevent—the progression of T2DM and its complications at the prediabetic phase. Therefore, the primary objective of this study was to determine whether a short-term intervention with SAIDEs for 6 months reduces blood glucose levels and inflammatory and immune markers at the end of the intervention or 6 months later. Additionally, we aimed to assess whether this short-term treatment could serve as a novel strategy for long-term diabetes prevention and management in prediabetic patients.

## 2 Methods

### 2.1 Study design and participants

**Figure 1:**
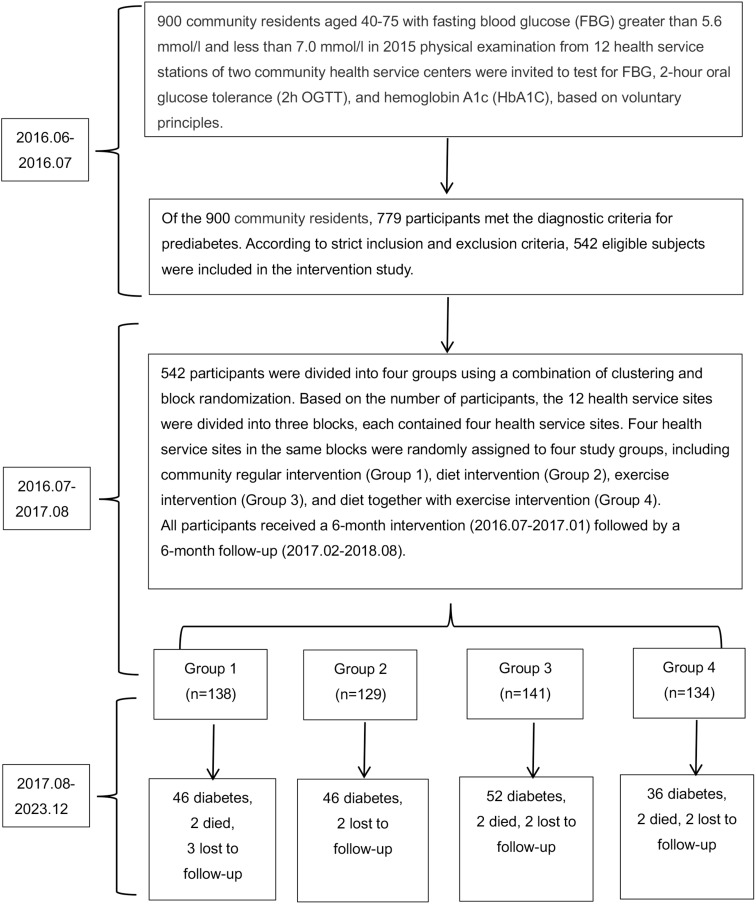
Study scheme.

We designed a prospective quasi-experimental study to evaluate whether SAIDEs could delay or prevent the onset of T2DM in Chinese adults with prediabetes. Additionally, we assessed both short- and long-term effects of SAIDEs on diabetes progression. In June 2016, we analyzed blood glucose markers in 900 prediabetic individuals aged 40–75 years, whose fasting blood glucose (FBG) levels were between 5.6 mmol/L and 7.0 mmol/L. These measurements were obtained from their 2015 physical examinations at 12 health service stations affiliated with two community health service centers in Huzhou, Zhejiang Province, China. The tested diabetic markers included FBG, 2-h oral glucose tolerance (2h OGTT), and hemoglobin A1c (HbA1C). Information on diabetes mellitus and medication use was also recorded. Among the 900 prediabetic individuals identified in 2015, 779 met the prediabetes criteria in June 2016. However, only 542 eligible prediabetic volunteers participated in the study (**Figure 1**).

### 2.2 Patient diagnosis, inclusion, and exclusion criteria

Diabetes and prediabetes were diagnosed according to the American Diabetes Association 2010 (ADA 2010) criteria or HbA1c-based diagnostic criteria (28–30). Diabetes was defined as FBG ≥ 7.0 mmol/L, 2h OGTT ≥ 11.1 mmol/L, or HbA1c ≥ 6.5%, whereas prediabetes was defined as FBG ≥ 5.6 and < 7.0 mmol/L, 2h OGTT ≥ 7.8 and < 11.1 mmol/L, or HbA1c ≥ 6.0% and < 6.5%. Normal individuals were defined as having a normal glucose profile if FBG < 5.6 mmol/L, 2h OGTT < 7.8 mmol/L, and HbA1c < 6.0%. Study participant inclusion criteria included: (1) meeting the diagnostic criteria for prediabetes; (2) no severe hypertension (systolic blood pressure [SBP] ≥ 180 mmHg or diastolic blood pressure [DBP] ≥ 110 mmHg), heart or brain diseases, or retinopathy; (3) not taking hypolipidemic drugs; (4) no serious liver or kidney defects; (5) no malignant tumors or other essential organ diseases; and (6) agreement to participate in the study. Study participant exclusion criteria included: (1) individuals diagnosed with diabetes who were taking hypoglycemic medications; (2) those with diabetes-related complications; (3) those unable to participate in the full-course intervention and follow-up. The numbers of patients included and excluded from all 12 health service stations are listed in **Supplementary Table S1** and **Figure 1**.

### 2.3 Sample size estimation for cluster randomization

Based on results from previous intervention studies (5–7), if 10% of participants in the community routine intervention (control group) achieve normal status, compared to 20% in the dietary or exercise intervention groups and 25% in the combined diet and exercise intervention group after 1 year, the test power is 80% (β = 0.20) with a type 1 error risk (α) of 0.05. Using the cluster random sampling sample size calculator (http://powerandsamplesize.com/Calculators/Compare-k-Proportions/1-Way-ANOVA-Pairwise), at least 476 participants were required for the 1-year intervention and follow-up study. Based on this calculation, we included 542 eligible prediabetic participants in this intervention study (**Figure 1**).

### 2.4 Study participant randomized grouping

A total of 542 qualified prediabetic participants were assigned to four groups using a combination of clustering and block randomization (5, 31). To ensure a similar number of participants at each site, three blocks were formed. Within each block, four sites were randomly assigned to one of the research groups: the community regular intervention group (group 1), the diet intervention group (group 2), the exercise intervention group (group 3), and the diet combined with exercise intervention (SAIDEs) group (group 4) (**Figure 1**, **Supplementary Table S1**). The questionnaires, physical examination assessments, and laboratory test items for intervention participants at baseline, during the intervention, and at follow-up are presented in **Supplementary Table S2**.

### 2.5 Basic information assessment

The following aspects were assessed using a self-developed questionnaire, the “Prediabetes Intervention Baseline Questionnaire”: (1) General demographic characteristics, including age, gender, place of residence, education level, work status, occupational status, and family income. (2) Major health history, covering blood pressure, blood lipids, blood sugar, heart disease, inflammatory and allergic diseases, as well as medication history (e.g., hypertensive drugs, hypolipidemic drugs, hypoglycemic agents, anti-inflammatory or antiallergic drugs for other conditions) and family history of the disease. (3) Lifestyle factors such as smoking, alcohol consumption, and sleep habits, along with physical examination data, including height, weight, blood pressure, and heart rate.

### 2.6 Dietary assessment

A standardized dietary assessment questionnaire was used to continuously record participants’ 3-day diet through self-reporting, assessing the intake of the three major energy substances (carbohydrates, proteins, and fats) as well as alcohol (32).

### 2.7 Physical activity status, exercise capacity, and risk assessment

The main assessment tools included both self-assessment and professional assessment for preexercise risk evaluation. Self-assessment involved completing a physical activity preparation questionnaire, while trained professionals conducted a professional assessment of participants’ cardiopulmonary function, metabolic disease risk factors, and symptoms or signs. This assessment determined whether participants required exercise, exercise under medical supervision, or exercise testing. The evaluation model used was the “Decision tree model for risk assessment of exercise adverse events in prediabetes” (33). Daily physical activity was assessed using the modified Total Energy Expenditure Questionnaire-Chinese version (TEEQ-C) (34) to measure participants’ exercise and nonexercise energy expenditure throughout the study period. The TEEQ-C assessment recorded participants’ activities over a 24-h period each week. Physical activity was evaluated using physical diaries, as previously reported (35) to enhance the understanding of participants’ activity levels and ensure accurate energy expenditure estimation. Trained investigators conducted the physical activity assessments. Weekly physical activity was calculated using the following formula: total metabolic equivalent (MET)-minute = item 1 (MET value × time (min/day) day/week) + item 2 (MET value × time (min/day) day/week) +… + item *n* (MET value × time (min/day) day/week).

### 2.8 Detection of biochemical indicators

Laboratory biochemical indicators included FBG, 2h OGTT, HbA1C, blood lipid profile (total cholesterol [TC], triglyceride [TG], high-density lipoprotein-cholesterol [HDL-C], and low-density lipoprotein-cholesterol [LDL-C]), insulin, and inflammatory and immune markers (hs-CRP, IL-6, TNF-α, IL-4, IL-10). FBG and 2h OGTT were measured using a Roche active blood glucose meter, all other biochemical indicators were measured at Hangzhou Di-An Biological Monitoring Company (Hangzhou, China). HbA1c was detected using an immunoturbidimetric assay with an automatic biochemical analyzer (C501, Roche Diagnostics, Indianapolis, IN). Insulin and C peptides were detected using electrochemiluminescence analysis with an electrochemiluminescence analytical instrument (E601, Roche Diagnostics). Blood lipid profile was analyzed using a biochemical method with an automatic biochemical analyzer (C501, Roche Diagnostics). Hs-CRP was measured using an enzyme-linked immunosorbent assay with an automatic biochemical analyzer (C501, Roche Diagnostics). Total IgE was assessed using immunoturbidimetric assay with a specific protein analyzer (Siemens BNP, Munich, Germany). TNF-α, IL-4, IL-6, and IL-10 were measured using the luminescence method with a luminescent instrument (DPC1000, Siemens, Washington, D.C, USA).

### 2.9 Study intervention groups

Group 1 was the control group, where participants received routine management from local community physicians without providing intervention materials. Group 2 was the dietary intervention group, with each participant receiving a self-compiled dietary intervention manual (DIM) and dietary intervention cards (DIC). Group 3 was the exercise intervention group, with each participant receiving a self-compiled prediabetes exercise intervention manual (EIM) and an exercise intervention card with exercise points. The main contents of DIM and EIM are shown in **Supplementary Table S3**. Participants in group 4 received both dietary and exercise interventions (SAIDEs). For those who received either or both interventions, standardization and consistency helped ensure health benefits. However, during the implementation process, we made minor adjustments to participants’ existing dietary and exercise habits.

In this study, the main contents of DIC included: (1) analyzing participants’ current dietary problems and emphasizing the health benefits of a reasonable and balanced diet; (2) introducing the recommended amount and proportions of the three daily macronutrients (carbohydrates, fats, and proteins) using food models and tools; (3) mapping the proper consumption of anti-inflammatory and antioxidant plant-based food; (4) presenting the glycemic index and glycemic load of common foods, guiding participants to select foods with low to moderate values, and highlighting the impact of cooking methods on these indices; and (5) outlining the key components of Chinese green tea (such as tea polyphenols, catechins, caffeine, and vitamin C), its benefits (including anti-inflammatory, antioxidant, mood regulation, and immunity enhancement), and recommended consumption practices (such as optimal drinking times). The course design of DIM was based on the “Diabetes Lifestyle Intervention Manual” (36) and the “Dietary Guidelines for Chinese Residents” (37). **Supplementary Table S3** provides detailed content on DIM. The exercise intervention included: (1) scientific exercise methods, emphasizing a combination of aerobic exercise and strength training, and highlighting the health benefits of public square dancing (such as improved heart and lung function, enhanced muscle strength and flexibility, better balance and coordination, calorie burning, weight control, stress relief, and mood enhancement) along with necessary precautions (such as exercising at appropriate times, selecting suitable venues and staying within one’s physical capacity); (2) weekly exercise routines, covering exercise type, frequency, and estimated total duration; (3) rest and recovery, focusing on preventing overtraining and exercise-related injury; and (4) balancing diet and exercise by reinforcing appropriate timing for meals and physical activity.

During the 6-month intervention period, intervention team members conducted group-based health education in the patients’ communities every 15 days: (1) group 2 (the dietary intervention group) received a total of six dietary interventions over 3 months, followed by monthly follow-ups for the next 3 months; (2) group 3 (the exercise intervention group) received a total of six exercise interventions over 3 months, followed by monthly follow-ups for the next 3 months; and group 4 (the SAIDEs group) received a total of 12 interventions over 6 months. Medical students regularly supervised the participants after training throughout the 6-month intervention period. Medical students and research instructors were responsible for addressing any questions from participants during the intervention. **Supplementary Figure S1** presents participants’ energy needs and prescriptions for dietary and exercise interventions.

### 2.10 Outcomes

During the 6-month intervention and initial 6-month follow-up, the primary indicator of interest was the conversion rate of blood glucose, including FBG, 2h OGTT, and HbA1C. The secondary indicators of interest were inflammatory and immune markers, including hs-CRP, IL-6, TNF-α, IL-4, and IL-10 to monitor the anti-inflammatory effects of dietary and exercise interventions. The tertiary indicators of interest primarily included body weight, blood lipid series, and insulin levels. Homeostasis model assessment–insulin resistance (HOMA-IR) was calculated as FBG × fasting insulin/22.5. The main indicator to monitored in the long-term follow-up was diabetes occurrence, assessed through annual physical examination records and health insurance data. During the 7.5 years of follow-up after the initial 12-month intervention (6-month intervention and 6-month follow-up), we collected FBG, 2h OGTT, and HbA1C data from patients via community physical examinations and the health insurance system (**Supplementary Table S2**) to determine diabetes incidence and time of onset.

### 2.11 Follow-up and intervention compliances

In interventional studies, particularly nonpharmacological interventions, follow-up and intervention compliance are essential for ensuring study authenticity and test efficacy. Our follow-up study consisted of three phases: (1) phase 1—follow-up during the 6-month intervention period, as described in the Study intervention groups; (2) phase 2—follow-up after the 6-month intervention, primarily conducted through telephone calls and community visits to assess patient health status, including blood glucose and dietary and exercise habits, and to address any issues encountered during the intervention; (3) phase 3—long-term follow-up, involving continuous collection of patient FBG, 2h OGTT, and HbA1C data to monitor the incidence and onset time of diabetes through community physical examinations and the medical insurance system over a 7.5-year following after the initial 12-month intervention study.

To improve the adherence, we implemented the following measures. First, the primary implementers of the project were medical students who had undergone standardized training by school instructors to work in rural and underdeveloped regions. Upon graduation, they were expected to return to their local community health service stations to contribute to chronic disease management. Before participating in the program and delivering interventions to research participants, students completed 6 months of professional training in investigative research and diabetes preventive interventions. All participating students were certified as having basic competencies in diabetes management by school instructors. Given the shortage of community healthcare workers at the time, utilizing qualified medical students as intervention implementers was a key measure to ensure the study’s smooth progression and participant compliance. Second, all selected participants completed a preliminary questionnaire survey and underwent various tests before formal enrollment in the intervention program. Third, unlike drug interventions that require strict dosage and administration schedules, this nondrug interventional study allowed for relatively easier assessment of treatment adherence. Fourth, to enhance participant adherence, we monitored dietary intervention compliance by assessing total and proportional intake of the three macronutrients (carbohydrates, protein, and fat), consumption of anti-inflammatory and antioxidant-rich plant foods and carbohydrate intake based on low glycemic index and low glycemic load instructions. We monitored the exercise intervention by assessing weekly exercise practice, type, frequency, and total volume of exercises.

### 2.12 Statistical analysis

In the descriptive analysis of each comparison group, the mean ± standard deviation was used continuous data that followed a normal distribution; otherwise, the median and interquartile range were reported. Missing or incomplete data were imputed using within-group means. Numerical variables were analyzed using one-way ANOVA analysis, and multiple comparisons between groups were conducted using the Student–Newman–Keuls (SNK) method. Comparisons across three time points were performed using repeated measures ANOVA. The paired *t*-test and one-way ANOVA were used to compare inflammation and immune factors at baseline and after 6 months of intervention. Analysis of the abnormal rate of blood glucose markers within the intervention groups at three different time points was performed using the paired-sample proportion test. The reduction in abnormal rates of the three glucose markers at 6 months (sixth months) and at the 6-month follow-up (12th month) between study groups was analyzed using the summary independent samples *t*-test, with the significance adjusted to 0.008 using the Bonferroni method (α’ = 0.05/6 = 0.008). Follow-up analysis was conducted using time-dependent covariate Cox regression analysis, Kaplan–Meier survival analysis, and log-rank test. Data processing and analysis were performed using SPSS23 analysis software. Unless otherwise specified, the significance level (α) for other hypothesis tests was set at 0.05.

## 3 Results

### 3.1 Short-term dietary and exercise interventions improved energy and glucose metabolisms and insulin sensitivity

We first measured the baseline characteristics of all four groups of prediabetic patients: community regular intervention (group 1), dietary intervention (group 2), exercise intervention (group 3), and dietary combined with exercise interventions (SAIDEs, group 4). The primary analysis variables included age, gender, body weight, body mass index (BMI), blood pressure, blood lipids, blood glucose, β-cell function-related indicators, inflammatory and immune-related indicators, and energy intake. Most of these variables did not differ significantly among the groups. However, blood pressure (*p* < 0.01), blood lipid levels (TC and TG) (*p* < 0.05), and 2-h postprandial insulin levels (2h PI) (*p* < 0.05) showed significant differences between some groups (**Table 1**).

**Table 1:** Baseline characteristics of each intervention group.

Variables (mean ± SD)	Group 1^a^ (*n* = 138)	Group 2^a^ (*n* = 129)	Group 3^a^ (*n* = 141)	Group 4^a^ (*n* = 134)	*F*/*χ* ^2b^	*p*-value^*^
Age	55.50 ± 6.52	54.83 ± 8.72	55.20 ± 9.42	56.47 ± 5.48	1.193	0.312
Sex (male/female)	66/72	52/77	46/95	48/86	7.580	0.056
Weight (kg)	65.12 ± 7.54	65.47 ± 7.55	64.89 ± 7.61	63.99 ± 8.12	0.892	0.445
BMI (kg/m^2^)	25.81 ± 4.42	24.73 ± 3.92	25.17 ± 4.99	25.48 ± 5.00	1.401	0.242
Blood pressure						
SBP (mmHg)	142.98 ± 16.97	137.09 ± 16.91	136.23 ± 21.38	140.27 ± 17.13	3.946	0.008
DBP (mmHg)	79.59 ± 9.88	78.57 ± 8.75	78.68 ± 10.67	83.11 ± 10.39	6.130	< 0.001
Blood lipid						
TC (mmol/L)	5.09 ± 1.05	5.04 ± 1.09	4.94 ± 0.92	5.42 ± 1.78	3.690	0.012
TG (mmol/L)	1.10 ± 0.33	1.14 ± 0.31	1.17 ± 0.33	1.15 ± 0.32	5.020	0.002
LDL-C (mmol/L)	2.58 ± 0.70	2.52 ± 0.61	2.52 ± 0.68	2.58 ± 0.98	0.289	0.834
HDL-C (mmol/L)	1.22 ± 0.29	1.17 ± 0.57	1.00 ± 0.60	1.20 ± 0.55	1.475	0.220
Blood sugar						
FBG (mmol/L)	5.51 ± 0.89	5.55 ± 0.81	5.60 ± 0.84	5.69 ± 0.76	1.115	0.342
2h OGTT (mmol/L)	8.24 ± 1.75	8.42 ± 1.63	8.00 ± 1.62	8.03 ± 1.61	1.861	0.135
HbA1C (%)	5.27 ± 0.72	5.14 ± 0.65	5.21 ± 0.72	5.23 ± 0.74	0.738	0.530
Beta cell function-related indicators						
Fasting insulin (µIU/mL)	15.96 ± 6.35	16.22 ± 6.64	15.25 ± 6.72	16.66 ± 6.14	1.159	0.325
2 h insulin (µIU/mL)	82.34 ± 25.86	80.62 ± 29.54	75.71 ± 28.82	85.20 ± 28.97	2.737	0.043
HOMA-IR	3.92 ± 1.76	4.00 ± 1.74	3.80 ± 1.76	4.23 ± 1.68	1.524	0.207
Fasting C peptide (ng/mL)	1.91 ± 0.64	1.97 ± 0.63	1.89 ± 0.61	1.97 ± 0.62	0.567	0.637
2-h postprandial C peptide (ng/mL)	8.59 ± 2.80	8.88 ± 2.83	8.53 ± 2.73	8.88 ± 2.80	0.622	0.601
Inflammatory and immunological-related indicators						
IgE (IU/L)	62.51 ± 30.25	59.39 ± 26.66	61.73 ± 28.50	62.71 ± 28.25	0.376	0.771
Hs-CRP (mg/L)	8.30 ± 4.13	8.41 ± 4.90	8.44 ± 4.68	8.28 ± 5.19	0.039	0.990
IL-6 (ng/L)	51.44 ± 17.10	48.85 ± 16.55	51.41 ± 17.93	48.66 ± 16.70	1.107	0.346
TNF-α (pg/mL)	242.17 ± 56.82	238.71 ± 54.68	242.09 ± 55.76	247.85 ± 54.19	0.619	0.603
IL-4 (ng/L)	215.98 ± 21.18	216.16 ± 20.16	213.61 ± 19.86	213.75 ± 20.30	0.622	0.601
IL-10 (ng/L)	207.23 ± 37.50	203.04 ± 38.34	207.67 ± 37.60	201.29 ± 37.75	0.940	0.421
Physical activity and energy intake assessment						
Total physical activity (MET-min/week)	2,870.32 ± 497.48	2,849.89 ± 484.46	2,862.00 ± 516.44	2,839.57 ± 527.50	0.096	0.962
Total energy intake (kcal)	2,113.98 ± 224.95	2,093.51 ± 220.60	2,111.09 ± 230.58	2,103.05 ± 229.18	0.219	0.883
Carbohydrate (%)	52.68 ± 4.19	52.49 ± 4.41	52.94 ± 4.31	52.18 ± 4.34	0.751	0.522
Fat (%)	28.43 ± 3.26	28.63 ± 3.40	28.07 ± 3.67	28.42 ± 2.99	0.673	0.569
Protein (%)	12.93 ± 3.03	12.46 ± 2.76	12.82 ± 2.99	12.81 ± 2.90	0.654	0.581
Alcohol (%)	6.43 ± 5.40	6.78 ± 5.40	6.73 ± 5.14	5.60 ± 2.18	0.391	0.759

*BMI*, body mass index; *SBP*, systolic blood pressure; *DBP*, diastolic blood pressure; *TC*, total cholesterol; *TG*, triglyceride; *LDL-C*, low-density lipoprotein cholesterol; *HDL-C*, high-density lipoprotein cholesterol; *FBG*, fasting blood glucose; *2h OGTT*, 2-h oral glucose tolerance test; *HbA1C*, hemoglobin A1C; *HOMA-IR*, homeostasis model assessment of insulin resistance; *IgE*, immunoglobulin E; *Hs-CRP*, high-sensitivity C-reactive protein; *IL*, interleukin; *TNF*, tumor necrosis factor.

^*^ *p* < 0.05 indicates at least one comparison reached statistical significance.

a Group 1: community routine intervention; group 2: diet intervention; group 3: exercise intervention; group 4: diet with exercise intervention.

b Numerical variables were analyzed using one-way ANOVA analysis (*F*). Multiple comparisons between the two groups were performed using the SNK method (*F*). Sex was analyzed using *χ* ^2^ tests.

Repeated measures ANOVA was used to assess whether structured dietary intake and exercise interventions affected energy intake and metabolisms by comparing baseline values with those recorded after 6 months of intervention and another 6 months of follow-up. Total energy intake and the proportions of carbohydrates, fat, and alcohol intake were significantly reduced over time in each intervention group (*p* < 0.001), although no significant differences were observed between the intervention groups, and there was no interaction between time and treatment. Protein intakes showed no significant changes over time within each intervention group, between the groups, or interactions between time and treatment (**Table 2**). Total physical activity in all intervention groups increased during the first 6 months of intervention and remained elevated after 6 months of follow-up (*p* < 0.001). Significant differences were observed between the groups, along with significant interactions between time and treatments (*p* < 0.001). Short-term 6-month exercise alone or SAIDEs increased total physical activity (**Table 2**). Both body weight and BMI significantly decreased over time in each intervention group (*p* < 0.001), but no significant differences were observed between the groups, and there was no interaction between time and treatment (**Table 2**). Similarly, both SBP and DBP significantly decreased over time in each intervention group (*p* < 0.001), with a significant difference between the groups, although no interaction was found between time and treatment (**Table 2**). Regarding blood lipid levels, only total cholesterol showed a significant decrease over time in each intervention group (*p* < 0.001). There were significant differences between the groups (*p* = 0.019) and interactions between time and treatment (*p* = 0.023). Although time did not significantly affect blood HDL-C levels within each intervention group (*p* = 0.494), significant differences were observed between the groups (*p* = 0.002), and there was a significant interaction between time and treatment (*p* = 0.002) (**Table 2**).

**Table 2:** Effect of a 6-month intervention and a 6-month follow-up on changes in diabetes-related outcomes.

Study group	Variable			Treatment effect *F* value (*p*-value)	Time effect *F* value (*p*-value)	Time and treatment interaction *F* value (*p*-value)
	Baseline	6-Month intervention	6-Month follow-up			
Total energy intake (kcal) (mean ± SD)						
Group 1	2,113.98 ± 224.95	1,880.26 ± 236.00	1,893.78 ± 233.43	0.126 (*p* = 0.945)	151.486 (*p* < 0.001)	0.271 (*p* = 0.950)
Group 2	2,093.51 ± 220.60	1,890.84 ± 246.23	1,910.93 ± 250.37			
Group 3	2,111.09 ± 230.58	1,871.45 ± 227.56	1,884.25 ± 240.32			
Group 4	2,103.05 ± 229.18	1,881.81 ± 242.19	1,901.53 ± 238.65			
Carbohydrate (%) (mean ± SD)						
Group 1	52.68 ± 4.19	49.96 ± 3.03	49.77 ± 2.95	1.227 (*p* = 0.299)	94.364 (*p* < 0.001)	0.531 (*p* = 0.775)
Group 2	52.49 ± 4.41	47.98 ± 3.09	50.08 ± 2.88			
Group 3	52.94 ± 4.31	50.15 ± 2.91	50.33 ± 3.07			
Group 4	52.18 ± 4.34	52.22 ± 3.04	49.72 ± 3.00			
Fat (%) (mean ± SD)						
Group 1	28.43 ± 3.26	26.83 ± 4.41	25.25 ± 2.79	0.283 (*p* = 0.837)	133.519 (*p* < 0.001)	1.235 (*p* = 0.288)
Group 2	28.63 ± 3.40	27.58 ± 8.30	24.86 ± 7.82			
Group 3	28.07 ± 3.67	28.63 ± 6.39	26.19 ± 5.99			
Group 4	28.42 ± 2.99 ±	27.69 ± 5.99	27.86 ± 4.86			
Protein (%) (mean ± SD)						
Group 1	12.93 ± 3.03	13.12 ± 2.77	12.86 ± 2.82	0.498 (*p* = 0.684)	1.435 (*p* = 0.239)	0.516 (*p* = 0.796)
Group 2	12.46 ± 2.76	12.03 ± 2.95	13.99 ± 3.00			
Group 3	12.82 ± 2.98	13.08 ± 3.13	12.65 ± 2.94			
Group 4	12.81 ± 2.90	12.85 ± 2.92	13.54 ± 2.83			
Alcohol (%) (mean ± SD)						
Group 1	6.43 ± 5.40	10.20 ± 5.73	12.12 ± 5.33	0.348 (*p* = 0.764)	537.00 (*p* < 0.001)	0.779 (*p* = 0.585)
Group 2	6.78 ± 5.40	9.57 ± 6.11	10.07 ± 5.28			
Group 3	6.73 ± 5.14	9.25 ± 5.81	11.16 ± 4.88			
Group 4	5.60 ± 2.18	9.28 ± 5.75	12.89 ± 5.11			
Total physical activity (MET-min/week) (mean ± SD)						
Group 1	2,870.32 ± 497.48	3,154.02 ± 650.70	2,922.60 ± 593.75	50.979 (*p* < 0.001)	109.507 (*p* < 0.001)	15.474 (*p* < 0.001)
Group 2	2,849.89 ± 484.46	2,963.89 ± 657.17	2,874.53 ± 596.50			
Group 3	2,862.00 ± 516.44	3,783.52 ± 707.72	3,266.40 ± 706.51			
Group 4	2,839.57 ± 527.50	3,770.36 ± 771.90	3,276.81 ± 673.27			
Weight (kg) (mean ± SD)						
Group 1	65.12 ± 7.54	61.93 ± 7.11	61.87 ± 7.25	1.138 (*p* = 0.333)	512.00 (*p* < 0.001)	1,074.00 (*p* = 0.328)
Group 2	65.47 ± 7.55	62.29 ± 7.27	62.25 ± 7.10			
Group 3	64.89 ± 7.61	61.57 ± 7.23	61.44 ± 7.23			
Group 4	63.99 ± 8.12	60.74 ± 7.90	60.47 ± 7.64			
BMI (kg/m^2^) (mean ± SD)						
Group 1	24.70 ± 0.98	23.49 ± 0.97	23.47 ± 1.05	2.398 (*p* = 0.067)	2,230.424 (*p* < 0.001)	1.173 (*p* = 0.321)
Group 2	24.86 ± 0.85	23.66 ± 0.91	23.65 ± 0.87			
Group 3	24.80 ± 0.89	23.54 ± 0.92	23.48 ± 0.93			
Group 4	24.63 ± 1.00	23.37 ± 1.08	23.28 ± 1.00			
SBP (mmHg) (mean ± SD)						
Group 1	142.98 ± 16.97	129.02 ± 12.05	129.84 ± 11.31	3.708 (*p* = 0.012)	63.981 (*p* < 0.001)	2.572 (*p* = 0.222)
Group 2	137.09 ± 16.91	130.45 ± 12.36	129.53 ± 10.43			
Group 3	136.23 ± 21.38	130.43 ± 12.01	130.07 ± 12.37			
Group 4	140.27 ± 17.13	132.17 ± 11.99	132.52 ± 11.80			
DBP (mmHg) (mean ± SD)						
Group 1	79.59 ± 9.88	77.33 ± 9.95	75.33 ± 10.13	3.632 (*p* = 0.013)	15.415 (*p* < 0.001)	1.932 (*p* = 0.732)
Group 2	78.57 ± 8.75	77.46 ± 11.05	77.55 ± 10.61			
Group 3	78.68 ± 10.67	76.74 ± 10.39	76.83 ± 10.12			
Group 4	78.68 ± 10.67	77.72 ± 10.25	77.85 ± 10.57			
TC (mmol/L) (mean ± SD)						
Group 1	5.09 ± 1.05	4.07 ± 0.68	4.12 ± 0.68	2.952 (*p* = 0.019)	429.00 (*p* < 0.001)	2.614 (*p* = 0.023)
Group 2	5.04 ± 1.09	4.07 ± 0.67	4.03 ± 0.70			
Group 3	4.94 ± 0.92	3.93 ± 0.66	3.96 ± 0.65			
Group 4	5.42 ± 1.78	4.00 ± 0.75	3.99 ± 0.67			
TG (mmol/L) (mean ± SD)						
Group 1	1.10 ± 0.33	1.15 ± 0.13	1.14 ± 0.17	2.920 (*p* = 0.034)	0.603 (*p* = 0.547)	0.516 (*p* = 0.796)
Group 2	1.14 ± 0.31	1.13 ± 0.17	1.12 ± 0.30			
Group 3	1.17 ± 0.33	1.16 ± 0.22	1.17 ± 0.19			
Group 4	1.15 ± 0.32	1.19 ± 0.10	1.21 ± 0.11			
LDL-C (mmol/L) (mean ± SD)						
Group 1	2.58 ± 0.70	2.71 ± 0.68	2.64 ± 0.75	0.138 (*p* = 0.937)	5.973 (*p* = 0.003)	0.831 (*p* = 0.545)
Group 2	2.52 ± 0.61	2.58 ± 0.75	2.74 ± 0.61			
Group 3	2.52 ± 0.68	2.63 ± 0.71	2.76 ± 0.79			
Group 4	2.58 ± 0.98	2.65 ± 0.70	2.67 ± 0.64			
HDL-C (mmol/L) (mean ± SD)						
Group 1	1.22 ± 0.29	1.15 ± 0.25	1.10 ± 0.35	4.932 (*p* = 0.002)	0.558 (*p* = 0.494)	4.403 (*p* = 0.002)
Group 2	1.17 ± 0.57	1.14 ± 0.14	1.15 ± 0.19			
Group 3	1.00 ± 0.60	1.15 ± 0.31	1.12 ± 0.22			
Group 4	1.20 ± 0.55	1.15 ± 0.19	1.15 ± 0.30			
FBG (mmol/L) (mean ± SD)						
Group 1	5.51 ± 0.89	5.28 ± 0.66	5.11 ± 0.72	0.666 (*p* = 0.574)	95.063 (*p* < 0.001)	1.013 (*p* = 0.386)
Group 2	5.55 ± 0.81	5.14 ± 0.73	5.10 ± 0.73			
Group 3	5.60 ± 0.84	5.17 ± 0.68	7.21 ± 0.69			
Group 4	5.69 ± 0.76	5.01 ± 0.72^a^	5.09 ± 0.71			
2 h OGTT (mmol/L) (mean ± SD)						
Group 1	8.24 ± 1.75	7.77 ± 1.69	7.95 ± 1.79	0.149 (*p* = 0.930)	10.602 (*p* < 0.001)	1.636 (*p* = 0.134)
Group 2	8.42 ± 1.63	7.70 ± 1.60	7.64 ± 1.65			
Group 3	8.00 ± 1.62	7.78 ± 1.54	7.98 ± 1.95			
Group 4	8.03 ± 1.61	7.76 ± 1.79	7.91 ± 1.67			
HbA1C (mmol/L) (mean ± SD)						
Group 1	5.27 ± 0.72	5.06 ± 0.65	4.89 ± 0.70	0.771 (*p* = 0.152)	27.414 (*p* < 0.001)	1.757 (*p* = 0.152)
Group 2	5.14 ± 0.65	4.90 ± 0.68	4.91 ± 0.68			
Group 3	5.21 ± 0.72	4.81 ± 0.66	5.01 ± 0.66			
Group 4	5.23 ± 0.74	4.96 ± 0.68	5.02 ± 0.62			
Fasting insulin (µIU/mL) (mean ± SD)						
Group 1	15.97 ± 6.35	13.68 ± 4.74	13.97 ± 4.81	2.221 (*p* = 0.085)	34.59 (*p* < 0.001)	3.217 (*p* = 0.163)
Group 2	16.22 ± 6.64	14.30 ± 4.93	14.67 ± 5.24			
Group 3	15.25 ± 6.72	13.47 ± 5.38	13.89 ± 5.78			
Group 4	16.66 ± 6.14	14.59 ± 4.81	15.06 ± 5.26			
2-h postprandial insulin (µIU/mL) (mean ± SD)						
Group 1	82.34 ± 25.86	71.59 ± 23.97	80.17 ± 26.72	2.446 (*p* = 0.063)	19.997 (*p* < 0.001)	1.317 (*p* = 0.248)
Group 2	80.62 ± 29.54	76.73 ± 24.28	83.33 ± 26.31			
Group 3	75.71 ± 28.82	70.96 ± 24.22	78.00 ± 22.14			
Group 4	85.20 ± 28.97	73.72 ± 29.93	80.39 ± 24.61			
Fasting C peptide (ng/mL) (mean ± SD)						
Group 1	1.91 ± 0.64	2.37 ± 0.85	1.93 ± 0.67	0.068 (*p* = 0.098)	89.945 (*p* < 0.001)	0.500 (*p* = 0.795)
Group 2	1.97 ± 0.63	2.36 ± 0.90	1.90 ± 0.65			
Group 3	1.89 ± 0.61	2.34 ± 0.84	1.92 ± 0.58			
Group 4	1.97 ± 0.62	2.29 ± 0.85	1.94 ± 0.63			
2-h postprandial fasting C peptide (ng/mL) (mean ± SD)						
Group 1	8.59 ± 2.80	11.84 ± 4.22	9.56 ± 3.27	0.061 (*p* = 0.98)	149.373 (*p* < 0.001)	0.498 (*p* = 0.792)
Group 2	8.88 ± 2.83	11.81 ± 4.52	9.55 ± 3.25			
Group 3	8.53 ± 2.73	11.70 ± 4.22	9.63 ± 2.93			
Group 4	8.88 ± 2.80	11.45 ± 4,25	9.70 ± 3.19			
HOMA-IR (mean ± SD)						
Group 1	3.92 ± 1.76	3.20 ± 1.15	3.20 ± 1.25	1.559 (*p* = 0.198)	77.699 (*p* < 0.001)	0.592 (*p* = 0.719)
Group 2	4.00 ± 1.74	3.25 ± 1.16	3.35 ± 1.35			
Group 3	3.80 ± 1.76	3.10 ± 1.32	3.21 ± 1.11			
Group 4	4.23 ± 1.68	3.23 ± 1.14	3.43 ± 1.36			

a The comparison of groups at three different time points was analyzed using repeated-measures ANOVA. Group 1: community routine intervention; group 2: diet intervention; group 3: exercise intervention; group 4: diet together with exercise intervention.

*BMI*, body mass index; *SBP*, systolic blood pressure; *DBP*, diastolic blood pressure; *TC*, total cholesterol; *TG*, triglyceride; *LDL-C*, low-density lipoprotein cholesterol; *HDL-C*, high-density lipoprotein cholesterol; *FBG*, fasting blood glucose; *2h OGTT*, 2-h oral glucose tolerance test; *HbA1C*, hemoglobin A1C; *HOMA-IR*, homeostasis model assessment of insulin resistance.

Repeated measures ANOVA on blood glucose and β-cell function-related indicators showed that FBG, 2h OGTT, HbA1C, fasting insulin, 2h PI, fasting C peptide, 2-h postprandial C peptide, and HOMA-IR significantly decreased over time in each intervention group (*p* < 0.001). However, no significant differences were observed between the groups, and there were no interactions between time and treatment (**Table 2**). Together, our results indicate that structured dietary or exercise interventions over time reduced energy intake; increased physical activity; decreased body weight, blood pressure, and cholesterol levels; and improved glucose metabolism, insulin sensitivity, and β-cell function.

### 3.2 Short-term dietary and exercise interventions reduced systemic inflammation

It is expected that reduced energy intake, increased physical activity, improved glucose metabolism, and enhanced insulin sensitivity may be associated with reduced systemic inflammation and immune activity. Therefore, we measured and compared various proinflammatory and anti-inflammatory molecules and immunological factors, including IgE, hs-CRP, TNF-α, IL-6, IL-4, and IL-10, at baseline and after 6 months of structured dietary and exercise interventions. After 6 months, all tested blood proinflammatory molecule levels—including IgE, hs-CRP, TNF-α, and IL-6—were significantly reduced in dietary, exercise, and SAIDEs groups (all *p* < 0.01). Patients in the routine community intervention group also showed significantly reduced levels of IgE (*p* < 0.001), hs-CRP (*p* < 0.001), and TNF-α (*p* = 0.003) after 6 months of intervention. In contrast, blood levels of anti-inflammatory cytokines IL-4 and IL-10 significantly increased in all four intervention groups after 6 months of intervention (*p* < 0.001) (**Table 3**). When proinflammatory and anti-inflammatory molecule levels were compared among the four intervention groups, only IL-6 and TNF-α (*p* < 0.001) showed significant reductions after 6 months of dietary, exercise, and SAIDE interventions. For example, IL-6 levels were significantly lower in groups 2, 3, and 4 than in group 1 (*p* < 0.001). Similarly, TNF-α levels were also significantly reduced in groups 2 and 4 patients compared with those in group 1 (*p* < 0.001) (**Table 3**).

**Table 3:** Reductions in blood inflammatory and immune factors after a 6-month intervention.

Factor	Study group	Number (*N*)	Mean difference (95% CI)	Comparison of baseline—sixth month within the group^a^		Comparison between groups^b^	
				*t*	*p*-value	*F*	*p*-value
IgE (ng/mL)	Group 1	138	12.32 (5.32, 19.31)	3.484	0.001	0.940	0.420
	Group 2	129	9.25 (2.72, 15.77)	2.804	0.006		
	Group 3	141	14.09 (7.78, 20.39)	4.418	< 0.001		
	Group 4	134	16.88 (11.04, 22.72)	5.720	< 0.001		
Hs-CRP (ng/mL)	Group 1	138	3.09 (2.27, 3.92)	7.402	< 0.001	0.258	0.856
	Group 2	129	3.60 (2.60, 4.59)	7.174	< 0.001		
	Group 3	141	3.16 (2.30, 4.01)	7.331	< 0.001		
	Group 4	134	3.40 (2.49, 4.30)	7.432	< 0.001		
IL-6 (ng/mL)	Group 1	138	2.04 (2.17, 6.25)	0.957	0.340	8.082	< 0.001^c^
	Group 2	129	12.12 (8.40, 15.85)	6.435	< 0.001		
	Group 3	141	14.15 (10.33, 17.97)	7.321	< 0.001		
	Group 4	134	12.63 (8.91, 16.36)	6.715	< 0.001		
TNF-α (ng/mL)	Group 1	138	20.40 (7.05, 33.74)	3.023	0.003	8.612	< 0.001^c^
	Group 2	129	51.69 (41.67, 61.71)	10.209	< 0.001		
	Group 3	141	28.19 (14.73, 41.65)	4.140	< 0.001		
	Group 4	134	57.93 (46.74, 69.12)	10.241	< 0.001		
IL-4 (ng/mL)	Group 1	138	− 32.36 (− 38.39, − 26.34)	− 10.621	< 0.001	0.744	0.526
	Group 2	129	− 35.59 (− 42.05, − 29.13)	− 10.896	< 0.001		
	Group 3	141	− 37.52 (− 43.45, − 31.59)	− 12.506	< 0.001		
	Group 4	134	− 38.16 (− 43.80, − 32.53)	− 13.386	< 0.001		
IL-10 (ng/mL)	Group 1	138	− 20.25 (− 29.89, − 10.60)	− 4.151	< 0.001	0.809	0.489
	Group 2	129	− 28.56 (− 38.75, − 18.37)	− 5.546	< 0.001		
	Group 3	141	− 18.72 (− 28.09, − 9.34)	− 3.948	< 0.001		
	Group 4	134	− 24.82 (− 34.82, − 14.82)	− 4.910	< 0.001		

Group 1: community routine intervention; group 2: diet intervention; group 3: exercise intervention; group 4: diet plus exercise intervention.

*IgE*, immunoglobulin E; *Hs-CRP*, high-sensitivity C-reactive protein; *IL*, interleukin; *TNF*, tumor necrosis factor.

a Paired *t*-test.

b One-way ANOVA test.

c IL-6: compared with group 1, significant differences were observed in groups 2–4 (*p* < 0.001). TNF-α: compared with group 1, significant differences were observed in groups 2 and 4 (*p* < 0.001), groups 2 and 3 (*p* = 0.007), and groups 3 and 4 (*p* = 0.001).

### 3.3 Short-term dietary and exercise interventions reduced the abnormal rate of glucose metabolism

Changes in the abnormal rate of blood glucose metabolism indicators—FBG, 2h OGTT, and HbA1C—after structured dietary and exercise interventions helped assess the effects of these interventions on glucose metabolism and diabetes development. The abnormal rate was defined as the percentage of patients with abnormal FBG, 2h OGTT, or HbA1C among the total number of participants in each study group. We compared the changes in the abnormal rate of these three markers across all four intervention groups at baseline, after 6 months of intervention (sixth month), and after an additional 6-month follow-up (12th month). The paired-sample proportion test showed significant reductions in these glucose metabolism markers after 6 months of intervention, with most of these reductions remaining significant after the follow-up period (**Table 4**). When patients with normal FBG, 2h OGTT, and HbA1C at baseline—or with one, two, or three of these indicators abnormal at baseline—were considered, we obtained similar observations. A short-term 6-month intervention significantly reduced the abnormal rates of FBG, 2h OGTT, and HbA1C at both sixth- and 12th-month time points in patients who had two of these glucose metabolism indicators abnormal at baseline (**Table 4**). The use of a summary independent samples *t*-test followed by Bonferroni adjustment (*α*’ = 0.05/6 = 0.008), allowed comparisons among different treatment groups to assess whether structured dietary and exercise interventions affected the abnormal rate of FBG, 2h OGTT, and HbA1C at different time points. Compared to patients who received community regular intervention (group 1), those who received dietary intervention (group 2), exercise intervention (group 3), or SAIDEs (group 4) showed varying degrees of reduction in the abnormal rates of FBG and HbA1C after 6 months of intervention (**Table 4**). Patients with two or three abnormal glucose metabolism indicators at baseline showed exhibited reduced abnormal rates after 6 months of intervention, with some reductions persisting after 6 months of follow-up (**Table 4**).

**Table 4:** Changes in the abnormal rate of blood glucose indicators (*n* [%]) at baseline, 6 months, and 12 months in each intervention group.

Study group^a^	*n*	Baseline	Sixth month^b^	12th month^b^
FBG				
Group 1	138	69 (50.0)	54 (39.1)	42 (30.4)^B^
Group 2	129	64 (49.6)	37 (28.7)^A,§1^	34 (26.4)^B,∮1,∯1^
Group 3	141	78 (55.3)	42 (29.8)^A,§2,§4^	48 (34.0)^B,∮2,∮4,∯2,∯4^
Group 4	134	81 (60.4)	33 (24.6)^A,§3,§5,§6^	39 (29.1)^B,∮3,∮5,∯3,∯5^
2h OGTT				
Group 1	138	89 (64.5)	70 (50.7)^A^	64 (46.4)^B^
Group 2	129	85 (65.9)	55 (42.6)^A,§1^	53 (41.1)^B,∮1,∯1^
Group 3	141	80 (56.7)	70 (49.6)^§2,§4^	70 (49.6)^∮2,∮4,∯2^
Group 4	134	78 (58.2)	68 (50.7)^§3,§5^	57 (42.5)^B,∮3,∮5,∮6,∯3,∯5,∯6^
HbA1C				
Group 1	138	61 (44.2)	47 (34.1)	38 (27.5)^B^
Group 2	129	45 (34.9)	37 (28.7)^§1^	37 (28.7)^∮1,∯1^
Group 3	141	58 (41.1)	40 (28.4)^A,§2,§4^	47 (33.3)^∮2,∮4,∯2,∯4^
Group 4	134	60 (44.8)	45 (33.6)^§3,§5,§6^	43 (32.1)^B,∮3,∮5,∮6,∯3,∯5,∯6^
All normal (FBG, 2h OGTT, HbA1C)^c^				
Group 1	138	0 (0)	31 (22.5)^A^	32 (23.2)^B^
Group 2	129	0 (0)	36 (27.9)^A,§1^	39 (30.2)^B,∮1,∯1^
Group 3	141	0 (0)	37 (26.2)^A,§2,§4^	32 (22.7)^B,∮2,∮4,∯4^
Group 4	134	0 (0)	31 (23.1)^A,§5,§6^	44 (32.8)^B,∮3,∮5,∮6,∯3,∯5,∯6^
One abnormal (FBG, 2h OGTT, HbA1C)^c^				
Group 1	138	66 (47.8)	54 (39.1)	71 (51.4)^C^
Group 2	129	73 (56.6)	65 (50.4)^§1^	61 (47.3)^∮1,∯1^
Group 3	141	75 (53.2)	64 (45.4)^§2,§4^	64 (45.4)^∮2,∮4,∯2,∯4^
Group 4	134	66 (49.3)	67 (50.0)^§3,§5,§6^	48 (35.8)^C,∮3,∮5,∮6,∯3,∯5,∯6^
Two abnormal (FBG, 2h OGTT, HbA1C)^c^				
Group 1	138	63 (45.7)	42 (30.4)^A^	32 (23.2)^B^
Group 2	129	47 (36.4)	20 (15.5)^A,§1^	24 (18.6)^B,∮1,∯1^
Group 3	141	56 (39.7)	32 (22.7)^A,§2,§4^	34 (24.1)^B,∮2,∮4,∯2,∯4^
Group 4	134	53 (39.6)	29 (21.6)^A,§3,§5^	35 (26.1)^B,∮3,∮5,∮6,∯3,∯5,∯6^
Three abnormal (FBG, 2h OGTT, HbA1C)^c^				
Group 1	138	9 (6.5)	11 (8.0)	3 (2.2)^C^
Group 2	129	9 (7.0)	8 (6.2)^§1,§4^	5 (3.9)^∮1,∯1^
Group 3	141	10 (7.1)	8 (5.7)	11 (7.8)^∮2,∮4,∯2^
Group 4	134	15 (11.2)	7 (5.2)^§3,§5,§6^	7 (5.2)^∮3,∮5,∮6,∯3,∯5,∯6^

Analysis of the abnormal rate of blood glucose markers within the intervention groups at three different time points was performed using a paired-sample proportion test. Analysis of abnormal rate reduction of glucose markers at 6 months (sixth month) and 6 months follow-up (12th month) between study groups used summary independent samples t-test and the test was adjusted to 0.008 by the Bonferroni method (*α*’ = 0.05/6 = 0.008).

a Group 1: Community routine intervention; Group 2: Diet intervention; Group 3:Exercise intervention; Group 4: Diet with exercise intervention.

b Sixth month: after 6 months of intervention; 12th month: after 6 months of intervention followed by another 6 months of follow-up.

c All normal: three items FBG, 2h OGTT, and HbA1C were normal; one abnormal: only one item in FBG, 2h OGTT, or HbA1C was abnormal; two abnormal: any two items FBG, 2h OGTT, or HbA1C were abnormal; three abnormal: all three items FBG, 2h OGTT, and HbA1C were abnormal.

^A–C^Comparisons within the same group between different time points: ^A^ *p* < 0.05 (baseline vs. sixth month); ^B^ *p* < 0.05 (baseline vs. 12th month); ^C^ *p* < 0.05 (sixth month vs. 12th month).

^§1–§6^Comparisons between the baseline value and that of the sixth month after intervention. ^§1^ *p* < 0.008 (group 1 vs. group 2); ^§2^ *p* < 0.008 (group 1 vs. group 3); ^§3^ *p* < 0.008 (group 1 vs. group 4); ^§4^ *p* < 0.008 (group 2 vs. group 3); ^§5^ *p* < 00.008 (group 2 vs. group 4); ^§6^ *p* < 0.008 (group 3 vs. group 4).

^∮1–∮6^Comparisons between the baseline value and that of the 12th month after the intervention. ^∮1^ *p* < 0.008 (group 1 vs. group 2); ^∮2^ *p* < 0.008 (group 1 vs. group 3); ^∮3^ *p* < 0.008 (group 1 vs. group 4); ^∮4^ *p* < 0.008 (group 2 vs. group 3); ^∮5^ *p* < 00.008 (group 2 vs. group 4); ^∮6^ *p* < 0.008 (group 3 vs. group 4).

^∯1–∯6^Comparisons between the result from the sixth-month intervention and that of the 12th-month intervention. ^∯1^ *p* < 0.008 (group 1 vs. group 2); ^∯2^ *p* < 0.008 (group 1 vs. group 3); ^∯3^ *p* < 0.008 (group 1 vs. group 4); ^∯4^ *p* < 0.008 (group 2 vs. group 3); ^∯5^ *p* < 0.008 (group 2 vs. group 4); ^∯6^ *p* < 0.008 (group 3 vs. group 4).

Together, the results from both the paired-sample proportion test and the summary independent samples *t*-test unequivocally demonstrate that structured dietary and exercise interventions reduced abnormalities in blood glucose metabolism markers—FBG, 2h OGTT, and HbA1C—both after the completion of the intervention treatment and after 6 months of follow-up.

### 3.4 Short-term dietary and exercise interventions delayed long-term diabetes onset

In addition to assessing the beneficial effects of the constructed dietary intervention, exercise intervention, or both (SAIDEs) on diabetes-associated energy intake reduction, physical activity increase, body weight loss, blood lipid level changes, and glucose metabolism and β-cell function improvements, we tested the possibility that the beneficial effects of short-term dietary intervention, exercise intervention, or both (SAIDEs) may also display long-term efficacy. We analyzed the incidence of diabetes over 8 years following the initial 6-month intervention treatments. During the 8-year follow-up, 46 prediabetic patients in group 1 developed diabetes, two patients died, and three were lost to follow-up in this group. Similarly, 46 prediabetic patients in group 2 developed diabetes, and two were lost to follow-up in this group. In group 3, 52 prediabetic patients developed diabetes, two died, and two were lost to follow-up. In group 4, 36 prediabetic patients developed diabetes, two died, and two were lost to follow-up (**Figure 1**). The diabetes incidence rates in the four groups were 33.33% (46/138) (group 1), 35.66% (46/129) (group 2), 36.88% (52/141) (group 3), and 26.87% (36/134) (group 4), which were not significantly different (*χ* ^2^ = 3.64, *p* = 0.303).

From the baseline analysis, we found that blood pressure (SBP, DBP), blood lipids (TC, TG), and 2h PI varied among the intervention groups (**Table 1**). Blood glucose levels often change with age. Therefore, we adopted the time-dependent covariate Cox regression analysis to test whether dietary and exercise interventions affected the incidence of diabetes and whether age, SBP, DBP, TC, TG, 2h PI, and gender were possible confounders to influence the effect of short-term interventions on diabetes incidence. However, our results showed that the diabetes incidence in prediabetic patients who underwent dietary intervention (group 2), exercise intervention (group 3), or SAIDEs (group 4) did not differ significantly from that of prediabetic patients in the community regular intervention (group 1) (**Table 5**).

**Table 5:** Time-dependent Cox regression analysis of diabetes incidence over an 8-year follow-up period.

	B	SE	Wald	*df*	Sig.	Exp(*B*)	95.0% CI for Exp(*B*)	
							Lower	Upper
T_COV	− 0.019	0.015	1.603	1	0.206	0.981	0.953	1.010
Group			4.171	3	0.244			
Group (1)	− 0.049	0.212	0.054	1	0.817	0.952	0.628	1.443
Group (2)	0.016	0.208	0.006	1	0.939	1.016	0.676	1.528
Group (3)	− 0.399	0.229	3.045	1	0.081	0.671	0.428	1.050
Gender	− 0.014	0.154	0.009	1	0.926	0.986	0.729	1.333
SBP	0.000	0.004	0.010	1	0.922	1.000	0.992	1.009
DBP	− 0.004	0.008	0.234	1	0.629	0.996	0.981	1.012
TC	0.077	0.062	1.551	1	0.213	1.080	0.957	1.219
TG	− 0.180	0.229	0.618	1	0.432	0.836	0.534	1.308
2h PI	− 0.001	0.003	0.199	1	0.655	0.999	0.994	1.004
Age	0.039	0.021	3.535	1	0.060	1.039	0.998	1.082

The group represents the main analysis factor, while the remaining factors serve as major adjustment factors. Groups (1) to (3) refer to group 2 (diet intervention), group 3 (exercise intervention), and group 4 (diet together with exercise intervention), with group 1 (community routine intervention) as the reference for analyzing the influence of different interventions on the incidence of diabetes.

*T_COV*, age as a time-dependent covariate; *SBP*, systolic blood pressure; *DBP*, diastolic blood pressure; *TC*, total cholesterol; *TG*, triglyceride; *2h PI*, 2-h postprandial insulin.

**Figure 2:**
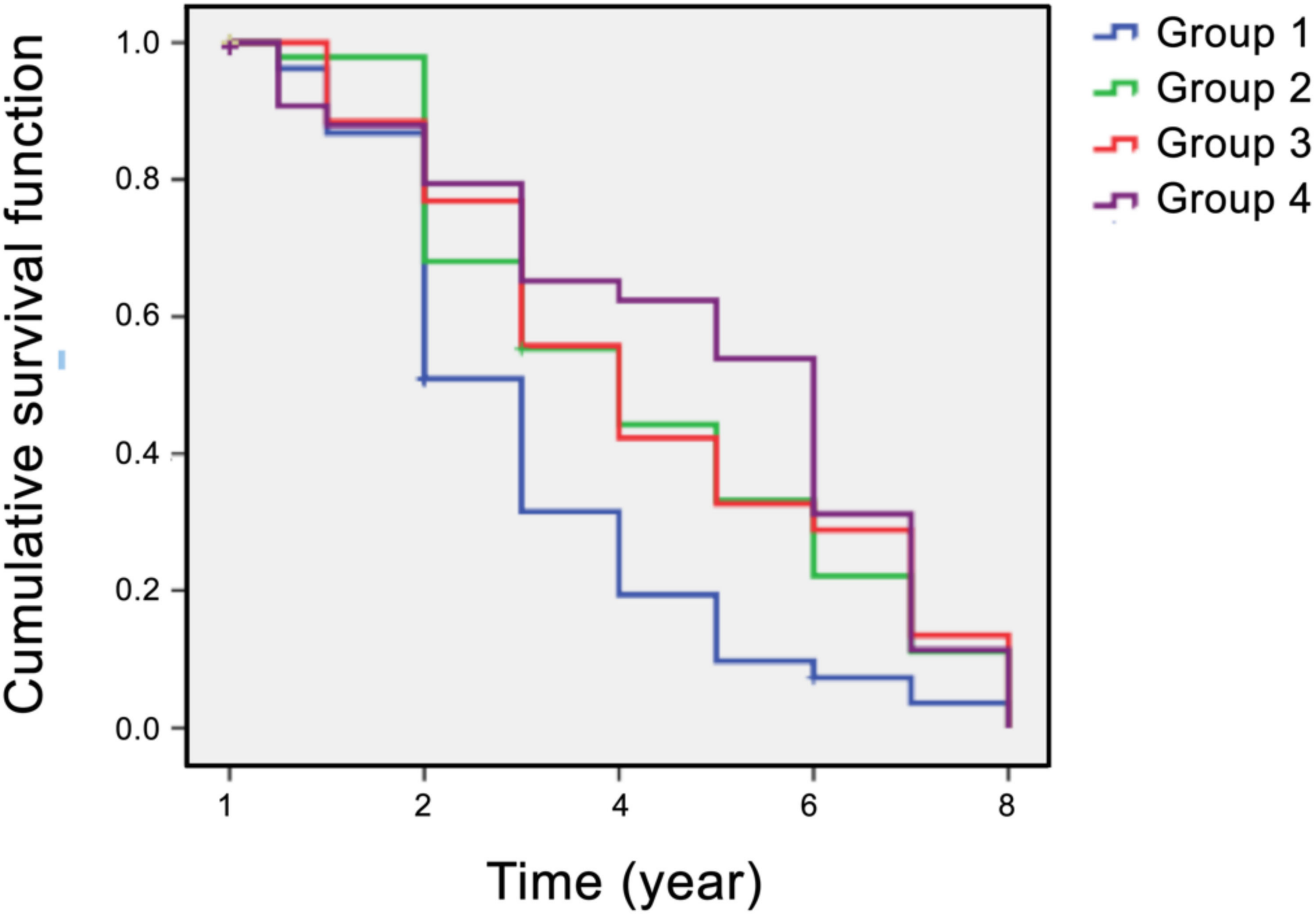
Kaplan–Meier plots of survival analysis (diabetes incidence) in all four participant groups over an 8-year follow-up period. Group 1: community routine intervention; group 2: diet intervention; group 3: exercise intervention; group 4: diet plus exercise intervention.

In addition to the time-dependent covariate Cox regression analysis, we performed Kaplan–Meier survival analysis to examine the distribution of mean survival time before the onset of diabetes in the four intervention groups. The results showed that the median “survival times” during the 8 years of follow-up were 3 years (95% CI: 2.5–3.5) in group 1, 4 years (95% CI: 2.7–5.3) in group 2, 4 years (95% CI: 3.0–5.0) in group 3, and 6 years (95% CI: 5.0–7.0) in group 4 (**Figure 2**). The log-rank test revealed statistically significant differences in the survival distribution among the four intervention groups (*χ* ^2^ = 15.63, *p* = 0.001). Compared to prediabetic patients who underwent short-term dietary intervention (group 2) and exercise intervention (group 3), the mean survival time before diabetes onset in those who receive from short-term SAIDEs (group 4) was significantly longer during the 8-year follow-up (**Figure 2**). Therefore, the 6-month short-term dietary intervention, exercise intervention, or SAIDEs all significantly delayed the disease onset during the 8-year follow-up, although these interventions did not reduce diabetes incidence.

## 4 Discussion

Our study demonstrated the beneficial effects of structured dietary intervention, with or without exercise, in patients with prediabetes. Even a short-term 6-month intervention reduced energy intake, intake, increased physical activity, and led to reductions in body weight, blood lipid levels, and blood glucose levels. These improved β-cell functions persisted not only after the completion of the intervention but also after an additional 6 months of follow-up (**Tables 2**-**4**). Although structured dietary intervention, with or without exercise, did not significantly reduce diabetes incidence over the 8-year follow-up period (**Table 5**), it delayed the onset of diabetes in prediabetic patients (**Figure 2**). While we lack solid evidence to fully explain these observations, it is well established that both prediabetes and diabetes are risk factors for cardiovascular diseases and *vice versa* (2–4). As individuals at high risk of prediabetes age, they inevitably progress to diabetes while also facing an elevated risk of cardiovascular diseases. Our findings suggest that intensive SAIDEs exert synergistic effects of diet and exercise interventions by reducing inflammation, blocking oxidative stress, and enhancing insulin sensitivity, which may serve as potential mechanisms for delaying the onset of diabetes (**Table 5**, **Figure 2**).

Chronic inflammation is primarily driven by endogenous substances or ligands released from tissue damage, which interact with pattern recognition receptors (PRRs) in the innate immune system. This interaction triggers excessive secretion of proinflammatory mediators by inflammatory cells. When chronic inflammation persists in blood vessels over an extended period, it contributes to vascular disease. Additionally, it can cause tissue degeneration and/or functional disorders in various organs, including endocrine tissues, and is recognized as a key factor in the development of cardiovascular diseases and diabetes (38). Ample evidence indicates that dietary and exercise interventions exert anti-inflammatory and antioxidant stress effects. The mechanisms underlying these benefits are multifaceted. Anti-inflammatory and antioxidant food components influence specific cell signaling pathways related to inflammation and oxidative stress, such as activating the Nrf2 pathway, reducing NF-κB signaling activity, increasing the NAD^+^ to NADH ration in mitochondria, and inhibiting histone deacetylase (HDAC) activity (39–45). The beneficial effects of anti-inflammatory and antioxidant diets include several key aspects. Certain anti-inflammatory antioxidant dietary components, such as vitamin E, polyphenols, brasses, and other antioxidants, help reduce the production of oxidants and inflammatory mediators (40–44). Omega-3 fatty acids found in certain anti-inflammatory foods can reduce the production of inflammatory mediators such as prostaglandin E2 (PGE2) and TNF-α (44). Additionally, dietary fibers can directly or indirectly strengthen the intestinal epithelial barrier, induce T-cell differentiation, and affect cytokine secretion, thereby mitigating the proinflammatory responses in the intestines and beyond (39, 45). Specific dietary patterns, such as the Mediterranean diet, emphasize the intake of plant-based foods rich in dietary fiber and various vitamins and minerals, which can help maintain gut health and promote the growth of beneficial microbial communities. These microbial communities produce short-chain fatty acids with anti-inflammatory effects (39, 45).

The mechanisms by which exercise exerts anti-inflammatory and antioxidant stress effects may include the following: exercise increases systemic production of anti-inflammatory factors and reduces the formation of inflammatory mediators. For example, clusterin is an exercise-induced plasma protein that can enter the brain through the bloodstream, reduce neuroinflammation, and improve memory (46). Exercise also reduces the production of proinflammatory factors such as TNF-α and IL-1β, thereby playing an anti-inflammatory role (38). Exercise not only improves endothelial function, promotes the synthesis of nitric oxide, lowers blood pressure, and reduces vessel wall inflammation but also enhances the resistance of vasculature against inflammation-induced damage through the activation of endogenous protective mechanisms by exercise-induced oxidative stress (47). Exercise increases energy expenditure and improves aging-related conditions, such as a decline in maximum oxygen uptake, oxidative stress, and various organ dysfunctions. The mechanism may involve the improvement of intracellular quality control systems (including the ubiquitin-proteasome system, autophagy, mitochondrial autophagy, and DNA repair) and reduction of endogenous ligand production, all of which help prevent the development of obesity, diabetes, dyslipidemia, and hypertension (38, 48). Exercise produces high levels of endogenous cannabinoids that mediate the production of anti-inflammatory substances by intestinal probiotics. Studies from patients with arthritis showed that serum levels of anti-inflammatory short-chain fatty acids including butyric acid, propionic acid, and isobutyric acid increased significantly after 6 weeks of muscle-strengthening exercises (49). Exercise stimulates the activity of antioxidant enzymes, such as superoxide dismutase (SOD) and catalase, and enhances the ability to remove free radicals, thereby exerting anti-inflammatory and antioxidant activity (50). Exercise improves mitochondrial dynamics, enhances mitochondrial antioxidant capacity, and improves the oxidative-reduction state of cardiac muscle (51). Exercise reduces visceral fat mass as a mechanism to reduce white adipocyte release of inflammatory molecules (52).

Many prior studies support the anti-inflammatory and antioxidant effects of diet and exercise interventions against prediabetes and the incidence and development of T2DM (5–7). However, the intervention components in these studies are relatively simple and lack structured and comprehensive approaches. Most of these studies evaluated only the effects of interventions on blood glucose, blood pressure, blood lipids, weight loss, energy intake, and exercise volume. Few studies have explored improvements in insulin activity, inflammation, and immune-related indicators from the perspective of pathological mechanisms (5–7). Our research explored the effects of diet and exercise on individuals with prediabetes using a structured and comprehensive intervention strategy. Unlike existing studies, our study had the following highlights and features: (1) Personalized plans, tailoring intervention strategies based on participants’ specific conditions (including dietary and exercise habits) to enhance adaptability and effectiveness; (2) Systematic design, ensuring that each stage of the intervention (including goal setting, implementation steps, and evaluation methods) followed a clear structure and process to maintain coordination and continuity; (3) Dietary intervention, emphasizing anti-inflammatory and antioxidant properties, primarily through the intake of carbohydrates with a low glycemic index load, as well as plant-based foods rich in dietary fiber, antioxidant vitamins C and E, and polyphenolic phytochemicals; and (4) Exercise intervention, incorporating both daily physical activities and structured exercise, including the use of fitness trails, sports facilities, and group square dancing in the surrounding community.

Nevertheless, study limitations remain: our research did not fully consider the differences in intervention effects among groups of different genders and different degrees of obesity, which might interfere with the interpretation of our results (53), although there were no significant differences in age, gender, body weight, and BMI among the four groups at the baseline (**Table 1**). However, based on the data from group 4 (SAIDEs), we performed the same analysis as in **Figure 2** and found there were no significant differences in the median “survival times” between male (6, 95% CI: 4.9–7.1 year) and female patients (5, 95% CI: 2.1–7.9 year) during the 8-year follow-up (*χ* ^2^ = 3.20, *p* = 0.073). This means that SAIDEs delayed the onset of diabetes independently of gender. It is also possible that Chinese patients in this age group (40~75 years old) generally have good compliance and healthy behaviors regardless of gender, a hypothesis that was not tested in this study. The assessment of dietary intake and physical activity has been a major challenge for epidemiological intervention studies. Self-report questionnaires used for assessment were susceptible to recall bias and measurement bias and may not have accurately quantify participants’ actual dietary and physical activity behaviors. For processing missing values, the “intra-group mean method” adopted in our study may be less robust than other methods, such as the “multiple filling method”. In this study, we focused only on rural residents aged 40–75. It remains untested whether the benefits of our intervention strategies and research outcomes can be extended to urban community residents, who typically have better lifestyles and Medicare benefits, or to individuals of other ages who may different levels of risks in diabetes development. China has a vast territory with diverse ethnic groups that exhibit significant differences in lifestyles. Our study targeted adults in a specific region (the Yangtze River Delta) in China, which may limit the generalizability of our findings. However, based on our previous results (36) and our current study, we proposed a structured anti-inflammatory lifestyle management model that we expect will generalize our findings to broader populations with different ethnic backgrounds and geographic regions. Therefore, there is an urgent need to conduct quantitative studies on the effectiveness of lifestyle interventions in slowing the progression of T2DM in patients with prediabetes, including those with IFG, IGT, or combined IFG and IGT.

## 5 Conclusion

Given the ongoing rise in the incidence and prevalence of diabetes worldwide, our study holds significant clinical value by integrating lifestyle changes into traditional diabetes interventions, even short-term modifications such as SAIDEs (described in this study). Both short-term and extended use of SAIDEs may contribute to effective public health measures for diabetes prevention. With sufficient government-led policy support, coordinated medical prevention efforts, effective community networking, and active individual participation in diabetes education and care, implementing SAIDEs could serve as an economical and effective measure to reduce diabetes prevalence and its related complications. These benefits may extend not only to prediabetic individuals but also to healthy and subhealthy populations.

## Data Availability

The raw data supporting the conclusions of this article will be made available by the authors, without undue reservation.
