# Supplementary material for: Short-term structured dietary and exercise interventions delay diabetes onset in prediabetic patients: a prospective quasi-experimental study
Source: Front Endocrinol (Lausanne). 2025 Mar 28;16:1413206. doi: 10.3389/fendo.2025.1413206 (PMC11985451; doi:10.3389/fendo.2025.1413206)
Supplement: Supplementary file 1 [file DataSheet1.pdf]

Supplementary Materials

Supplementary Table S1. The inclusion, exclusion, and grouping of participants in each community health service station.

Heath service stations	Target participants	Excluded	Reasons for exclusion				Participants included	Participant grouping
			Too high blood pressure	Use of lipid-lowering drugs	Major organ diseases	Other reasons		
**1**	61	15	5	4	3	3	46	4
**2**	72	30	9	6	5	10	42	3
**3**	75	25	9	5	4	7	50	1
**4**	72	13	4	5	3	1	59	1
**5**	88	24	5	6	7	6	64	4
**6**	48	19	6	6	4	3	29	1
**7**	108	27	8	7	6	6	81	3
**8**	32	8	2	2	2	2	24	4
**9**	30	12	4	3	2	3	18	3
**10**	28	4	2	1	1	0	24	2
**11**	20	4	1	0	1	2	16	2
**12**	145	56	5	6	6	39	89	2

Supplementary Table S2: List of questionnaires and test items involved during baseline, intervention, and follow-up.

Items	Baseline	6 months of intervention	Follow-up 6 months	Follow-up additional 7.5 years
**Clinical assessment**				
History of disease	●	●	●	
History of medication	●	●	●	
Physical examination	●	●	●	
Cardiovascular risk score	●	●	●	
Elements of the metabolic syndrome	●	●	●	
Blood pressure	●	●	●	
Height	●	●	●	
Weight	●	●	●	
**Detection of biochemical indicators**				
Fasting blood glucose (FBG)	●	●	●	●
2-hour oral glucose tolerance (2h OGTT)	●	●	●	●
Hemoglobin A1c (HbA1C)	●	●	●	●
Fasting insulin	●	●	●	
2 hours postprandial insulin	●	●	●	
Fasting C peptide	●	●	●	
2 hours postprandial C peptide	●	●	●	
Total cholesterol	●	●	●	
Triglyceride	●	●	●	
Low-density lipoprotein cholesterol	●	●	●	
High-density lipoprotein cholesterol	●	●	●	
Inflammatory and immune markers (Hs-CRP, IgE, TNF-α, IL-4, -6, -10)	●	●	●	
**Dietary and physical activity assessments**				
Dietary assessment	●	●	●	
Physical activity assessment	●	●	●	

Supplementary Table S3: Content summary of the diet and exercise intervention manuals.

Dietary intervention manual (DIM) group	Exercise intervention manual (EIM) group
1. Basic knowledge of diabetes mellitus (pre-diabetes mellitus)	1. Basic knowledge of diabetes mellitus (pre-diabetes mellitus)
2. Reasonable diet (preparation of dietary formulation)	2. The health benefits of exercise
3. Carbohydrate and blood glucose control	3. Anti-inflammatory and antioxidant effects of exercise
4. Lipids and blood glucose control	4. Exercise ability and exercise medicine assessment
5. Protein and blood glucose control	5. Principles of exercise prescription formulation
6. Vitamin and blood glucose control	6. Exercise prescription for common chronic diseases
7. Minerals and blood glucose control	7. Nutritional management of exercise
8. Antioxidant and anti-inflammatory diet and blood glucose control	8. The management of exercise-induced fatigue
9. Food allergy and blood glucose control	9. Prevention and management of sports injuries
10. Nutritional therapy with hyperglycemia associated with metabolic abnormalities	10. Develop and maintain an exercise program

Assessment of the energy intake requirements

↓

1. Calculation of basal metabolic rate (BMR)

Male: 10 x weight (kg) + 6.25 x height (cm) – 5 x age (year) + 5

Female: 10 x weight (kg) + 6.25 x height (cm) – 5 x age (year) – 161

↓

2. Estimated amount of total energy expenditure based on the BMR

People who sit for a long time or work in office work: BMR x 1.2

People with mild exercise intensity and who exercise 1-3 times a week: BMR x 1.375

People with moderate exercise intensity and who exercise 3-5 times a week: BMR x 1.55

People with high exercise intensity and who exercise 6-7 times a week: BMR x 1.725

People with extremely high exercise intensity and with a daily physical training or physical labor: BMR x 1.9

↓

3. Three major thermal nutrients supply ratio calculation

Carbohydrates: Fat: Protein =60%: 25%: 15% or

Carbohydrates: Fat: Protein =70%: 20%: 10%

↓

4. Three major thermogenic nutrients supply range (kg) calculation

Carbohydrate supply amount (kg) = carbohydrate supply of heat (kcal) / 4 (kcal/kg)

Fat supply amount (kg) = fat supply of heat (kcal) / 9 (kcal/kg)

Protein supply amount (kg) = protein supply of heat (kcal) / 4 (kcal/kg)

↓

5. Three major thermal nutrients range of food exchange portion calculation

The number of carbohydrate portion = carbohydrate supply of heat (kcal) /90 (kcal)

The number of fat portion = fat supply of heat (kcal) / (kcal)

The number of protein portion = protein supply of heat (kcal) (kg)/(kcal)

↓

6. Three energy nutrients meal requirements calculation

(30% for breakfast, 40% for lunch, dinner for 30%)

↓

7. Dietary guidelines

Guidelines for anti-inflammatory and antioxidant food consumption

(only for Group 2 and Group 4)

Quick check chart for common food the food exchange portion

Supplementary Figure S1. Energy intake requirement assessment and dietary prescriptions for dietary and exercise intervention participants. This study estimated total energy expenditure based on the participants' basal metabolic rate (BMR) combined with the participants' exercise intensity. Participant’s energy intake was determined according to the estimated energy expenditure. The estimated total energy expenditure is allocated according to a reasonable supply ratio of the three major thermogenic nutrients (carbohydrate, fat, protein) and meal habits. This study only provided anti-inflammatory and antioxidant dietary guidelines for participants in the diet intervention group (Group 2) and diet together exercise intervention group (Group 4).
